# The mechanisms linking work-life balance and well-being among Chinese college teachers

**DOI:** 10.3389/fpubh.2025.1602643

**Published:** 2025-05-20

**Authors:** Changwu Wei, Yan Ma, Jian-Hong Ye

**Affiliations:** ^1^Normal College, Hezhou University, Guangxi, China; ^2^Chinese International College, Dhurakij Pundit University, Bangkok, Thailand; ^3^School of Foreign Languages, Hezhou University, Guangxi, China; ^4^Faculty of Education, Beijing Normal University, Beijing, China

**Keywords:** job demands, work engagement, work-life balance, well-being, physical and mental health, interpersonal harmony, sense of achievement

## Abstract

**Introduction:**

College faculty well-being is crucial for higher education goals and sustainability. However, their well-being is lower than in other professions. High job demands in academia impede work-life balance, a key well-being determinant, while work engagement can counterbalance these demands. However, the mechanisms linking job demands, work engagement, work-life balance, and well-being remain underexplored.

**Methods:**

This study explores these mechanisms using the Work-Life Balance model. Semi-structured interviews were conducted with 13 Chinese college teachers, and thematic analysis was used to identify themes and construct a systematic model.

**Results:**

Perceived job value, family support, and financial security can reduce job demands and enhance work-life balance. Excessive demands, however, may overwhelm teachers, causing health issues and disrupting personal life. Job value, responsibility, work interest, and family support also boost work engagement, which, combined with rewards, improves balance. Financial security, family support, and social status are critical for achieving work-life balance, which is essential for health, interpersonal harmony, and overall well-being.

**Discussion:**

These findings reveal complex, non-linear relationships among job demands, work engagement, work-life balance, and well-being in Chinese higher education. They highlight the need for nuanced understanding and provide insights for policy and institutional reforms.

## 1 Introduction

The well-being of college faculty is critical to achieving the goals of higher education and ensuring its long-term sustainability (1). Well-being is defined as a positive psychological state characterized by contentment and pleasure, which arises from the fulfillment of needs and expectations within the constraints of reality (2). Despite its importance, research consistently indicates that college faculty report lower levels of well-being compared to other professions (3). This disparity is attributed to the unique challenges faced by academic professionals, including high job demands, emotional labor, and an intense work pace (4). Within the context of China’s ongoing educational reform, which emphasizes transformative practices and heightened performance expectations, college faculty members face multiple pressures (5). These professionals are tasked with fulfilling diverse roles, including teaching, research, and community service. Consequently, these overlapping responsibilities not only increase time and energy demands but also intensify work-related stress due to heightened performance expectations in research and community service (6, 7). Furthermore, socio-cultural contexts profoundly shape teacher behavior and performance. In China, teachers are often positioned as “social role models,” which amplifies the pressure for professional development (8). Additionally, the cultural emphasis on collectivism and familial obligations—such as elder care and child-rearing (9)—presents substantial challenges for faculty members striving to balance professional success with family life (10). When these multiple pressures are left unaddressed, they can lead to health issues and negatively impact overall well-being. Therefore, comprehending these dynamics is crucial for addressing the unique challenges encountered by Chinese college faculty members.

Work-life balance is a key determinant of well-being and involves the harmonious integration of professional and personal life domains with minimal conflict between them (11, 12). A balanced work-life interface not only enhances life satisfaction but also contributes to psychological well-being (13). Conversely, imbalances, particularly those stemming from work–family conflicts, can undermine well-being (14). For college faculty, achieving work-life balance is strongly correlated with overall well-being. Studies have reported positive associations between a balanced work-life interface and enhanced well-being among university teachers (15, 16). Conversely, imbalances in this population—often resulting from overlapping pressures of work and personal life—have been linked to negative outcomes, including reduced well-being (17, 18). These imbalances compress leisure time, accumulate negative emotions, and intensify work demands, thereby eroding teachers’ well-being (18). Notably, Ono et al. (17) found that high levels of work–family conflict significantly undermine the well-being of teachers aged 30–39 years. Similarly, Lin et al. (19) demonstrated that in China, work-life imbalance due to work–family conflicts is detrimental to college faculty well-being, whereas a supportive work-life interface fosters positive outcomes. Therefore, addressing these imbalances is crucial for enhancing the well-being of college faculty.

Despite the established importance of work-life balance, job demands inherent to the academic profession often create barriers to achieving equilibrium (20). Job demands encompass the physical, psychological, social, and organizational tasks associated with one’s role, which require sustained effort and skills and are linked to physiological and psychological costs (21). Empirical evidence indicates that faculty members typically experience moderate to high levels of job demands (22), and rising job demands have been shown to negatively predict work-life balance among academics (23, 24). This relationship may stem from heightened job demands leading to intrusive work-related thoughts, reduced engagement in leisure activities, or a diminished perception of work-life balance—all factors that can impair overall well-being (25).

While job demands pose challenges to work-life balance, work engagement serves as a counterbalance by facilitating equilibrium (26). Work engagement is defined as a positive, fulfilling state of vigor and dedication in which individuals willingly invest themselves in their professional responsibilities (27). Work engagement is a crucial factor for teachers in China, particularly for novice teachers, as suggested by Ye et al. (28). This finding aligns with the broader observation that college faculty generally exhibit high levels of work engagement (29). This may be attributed to the fulfillment of fundamental needs related to survival, happiness, and accomplishment (30). As a profession characterized by ethical consciousness, teaching involves interpersonal exchange, intellectual stimulation, knowledge sharing, and emotional interactions, all of which contribute to a sense of joy and accomplishment (69).

While prior research has documented the negative correlation between job demands and work-life balance, the positive correlation between work engagement and work-life balance, and the positive link between work-life balance and well-being, the underlying mechanisms driving these relationships remain underexplored. This study seeks to address this gap by examining the interplay among these variables through qualitative interviews with Chinese college faculty. By exploring the contributory factors and outcomes of these relationships, this study aims to elucidate how specific elements either diminish or enhance college faculty well-being. Furthermore, while quantitative research has provided valuable insights into these relationships (11, 20, 26), it falls short in explaining the nuanced reasons behind them. Qualitative methods are therefore essential to uncover the complex interconnections among these variables (31). Based on these objectives, the study addresses the following research questions:

- How do Chinese college faculty members perceive the relationship between job demands and work-life balance?
- How do Chinese college faculty members perceive the relationship between work engagement and work-life balance?
- How do Chinese college faculty members perceive the relationship between work-life balance and well-being?

## 2 Theoretical foundations and terminology definitions

### 2.1 Work-life balance model

The work-life balance model incorporates a variety of individual and organizational factors that influence work-life balance, along with the potential outcomes related to both work and non-work spheres (12). This model can be succinctly depicted as follows: antecedents → work-life balance → outcomes. Antecedents are comprised of individual factors, such as work engagement, self-efficacy, sense of responsibility, and time management abilities, etc., and organizational factors, including job demands and time pressure, among others. Outcomes encompass work-related dimensions like occupational well-being, job satisfaction, and organizational commitment as well as non-work-related aspects such as personal well-being, life satisfaction, and marital fulfillment.

Considering the plentiful potential antecedents and outcomes, it is impractical to enumerate them exhaustively. Therefore, according to the specific research purpose, researchers may select relevant antecedents and outcomes (12) or simplify the work-life balance model (32). This study employs the work-life balance model as its foundational framework, investigating the specific reasons for those associations between the variables, specifically, negative between job demands and work-life balance, positive between work engagement and work-life balance, and positive between work-life balance and well-being, from the standpoint of college faculty in China.

### 2.2 Job demand

Originally introduced within the Job Demand-Control Model (33), Job demand refers to the volume and complexity of tasks that engender stress, including workload and problem-solving requirements. Demerouti et al. (21) extended this notion, conceiving job demands as either physical or psychological requisites that a position imposes on an individual—essentially, the ongoing exertion of physical or psychological effort necessary for job completion, such as work stress, emotional demands, and interpersonal demands. Subsequently, building upon the concept of Demerouti et al. (21), scholars argued that job demands require employees to exert continuous physical and/or psychological effort (34). This present study adopts the definition of job demands as conceptualized by Demerouti et al. (21).

### 2.3 Work engagement

Work engagement evolves from the broader construct of personal engagement (35), which characterizes a state in which individuals are wholeheartedly absorbed in their tasks, including physiological, cognitive, and emotional involvement, indicating that individuals actively participate in tasks, maintain focus and attentiveness, and effectively express their thoughts and feelings. Subsequently, May et al. (36) proposed that work engagement consists of three dimensions: cognitive, emotional, and physical engagement. Furthermore, some scholars view work engagement as a positive, fulfilling, work-related state characterized by vigor, dedication, and concentration (37). Both concepts underscore the wholehearted engagement in work. In this study, work engagement is defined as a vibrant and focused work state, characterized by a willingness to contribute to work with energy and attentiveness.

### 2.4 Work-life balance

Work-life balance initially referred to a satisfied, healthy, and productive life that includes work, leisure, and relationships (38). Some scholars also view work-life balance as the equilibrium between different roles (39). Subsequently, work-life balance has primarily been explored in terms of conflicts (40), interference (41), facilitation (42), time balance (43), resource balance (44) between work and family. Some scholars view work-family balance as a subjective experience (45). Currently, scholars have expanded this concept from the domain of family to the broader domain of life, emphasizing the maximizing of work-life engagement and the minimizing of conflicts (12), and focusing on individuals’ sense of balance between work and non-work roles (46). In this study, work-life balance refers to the harmonious, coordinated, and balanced relationship between individuals’ work and life roles.

### 2.5 Well-being

The terms well-being and psychological well-being are often used interchangeably and are also interconnected in research (47). While scholars are not unanimous in the concept of well-being, their studies are primarily rooted in hedonic and eudaimonic perspectives (48). Based on the hedonic perspective, Diener (49) proposed the concept of subjective well-being, which encompasses life satisfaction, positive and negative emotions. From the eudaimonic perspective, Ryff (50) highlighted well-being as a positive psychological function. Waterman (2) integrated both perspectives and introduced the concept of psychological well-being. In this study, well-being refers to an individual’s positive psychological experience of contentment and joy when their needs are fulfilled and expectations are met within the constraints of reality.

## 3 Research methods

### 3.1 Selection of participants

Guest et al. (51) noted that in cases where participants are relatively homogeneous, 94% of high-frequency codes would emerge within the first six interviews, and 97% would appear within the first 12 interviews. Similarly, Ando et al. (52) suggested that 12 interviews are adequate to capture all necessary themes. To determine sample saturation, we utilized an encoding saturation table, which involves cross-tabulating codes and case entries (53). Codes that appeared in the first case were recorded, and new codes emerging in subsequent cases were documented until no new codes emerged. In this study, it was found that no new codes emerged in Cases 11, 12, and 13, indicating that data saturation had been reached and all themes were adequately represented. Thus, data collection could be terminated. Thematic Coding Saturation is shown in Table 1.

**Table 1:** Thematic coding saturation.

Code	Interviewee												
	1	2	3	4	5	6	7	8	9	10	11	12	13
JD → WLB													
Life Disturbance	√												
Job value		√											
Mental Health Issues			√										
Physical Health Issues	√												
Capacity Exceedance		√											
Family Support					√								
Financial security						√							
WE→WLB													
Job value	√												
Work Interest										√			
Work Rewards			√										
Sense of Achievement				√									
Job Responsibility					√								
Family Support						√							
WLB → WB													
Interpersonal Harmony	√												
Physical Health									√				
financial security		√											
Mental Health	√												
Social Status								√					
Family Support							√						

JD refers to job demands; WLB refers to work-life balance; WE refers to work engagement; WB refers to well-being.

We selected a sample of 13 participants using purposive sampling. This participant selection process was designed to ensure a balanced representation across different regions, gender, age, professional title, marital status, family size etc. Demographic details are shown in Table 2.

**Table 2:** Interviewee profile.

Participant	District	Gender	Age	Public/private	Professional title	Full/Part time	marital status	Number of children
M01	West	Male	≤35	Public	Intermediate	Part-time	Married	1
M02	Central	Male	≤35	Private	Intermediate	Part-time	Single	0
M03	Central	Male	36–45	Public	Intermediate	Full-time	Married	1
M08	West	Male	≥46	Public	Intermediate	Full-time	Married	2
M09	West	Male	≥46	Public	Intermediate	Full-time	Married	2
M10	West	Male	≤35	Public	Intermediate	Part-time	Single	0
M11	Central	Male	36–45	Private	Senior	Part-time	Married	2
F04	East	Female	≤35	Private	Intermediate	Full-time	Single	0
F05	Central	Female	≤35	Public	Senior	Full-time	Married	1
F06	East	Female	≤35	Public	Senior	Full-time	Married	1
F07	East	Female	≤35	Private	Intermediate	Part-time	Married	2
F12	West	Female	36–45	Private	Senior	Full-time	Married	2
F13	West	Female	36–45	Public	Senior	Full-time	Married	2

M01 denotes the first male teacher interviewed, F04 denotes the fourth female teacher interviewed, and so on.

### 3.2 Implementation of interviews

The interviews were conducted remotely via the Tencent Meeting platform and audio-recorded to facilitate subsequent coding and analysis. Participants were informed that they would be invited to review the findings to ensure accuracy through member checking. Although an interview guide was prepared, flexibility was maintained regarding the exact wording and sequence of questions to encourage a natural flow of conversation (54). Participants received the informed consent form and an outline of the interview 1 day prior to their scheduled session before the interview commenced. This study was ethically reviewed according to the procedures required by the Human Research Ethics Committee of Dhurakij Pundit University in Thailand (Certificate No. DPUHREC006/65EX).

### 3.3 Interview guide

The interview guide was systematically developed to align with the research objectives and underwent a rigorous validation process. Five education management Ph.D. experts with at least 5 years of teaching experience in higher education evaluated the guide. The evaluators assessed each interview question individually, categorizing them into three types: (1) retain without modification; (2) retain after modification; and (3) delete. For questions categorized as (2) or (3), detailed justifications were provided. After three rounds of evaluation and modification, the content validity of the interview schedule was ultimately established. This iterative process ensured that the final set of interview questions was both relevant and comprehensive, thereby enhancing the overall reliability and validity of the study. The main interview queries were constructed as follows:

- In your opinion, what factors increase the job demands for college faculty members? What factors can reduce the adverse effects of job demands on them? Why do you think so?
- What potential outcomes may result from job demands? How do these outcomes relate to work-life balance for college faculty members? Why do you think so?
- In your opinion, what factors increase or decrease the work engagement of college faculty members? Why do you think so?
- What potential outcomes may result from work engagement? How do these outcomes relate to the achievement of work-life balance for college faculty members? Why do you think so?
- In your opinion, what factors facilitate or hinder the achievement of work-life balance for college faculty members? Why do you think so?
- What potential outcomes may result from work-life balance? How do these outcomes relate to the promotion of well-being for university teachers? Why do you think so?

## 4 Results

In this study, thematic analysis was employed to analyze the interview data. It is an approach that facilitates identifying patterns within qualitative data, independent of any single theoretical framework, allowing for integration across different theoretical understandings (55). Following Morse et al. (56), our data analysis upheld methodological rigor, ensuring methodological consistency, sample appropriateness, concurrent data collection and analysis, theoretical thinking processes, and deliberate development of theoretical constructs, which underpinned the reliability and validity of the research findings. This analytical process yielded 12,691 Chinese characters, producing a comprehensive report comprising 99 concepts, 5 themes, and 19 subthemes, detailed in Table 3.

**Table 3:** Thematic analysis code.

Theme	Sub-theme	Frequency
Factors that alleviate job demand	Job value	6
	Family Support	7
	Financial security	6
Intermediary factors between job demand and work-life balance	Physical Health Issues	3
	Mental Health Issues	4
	Capacity Exceedance	4
	Life Disturbance	5
Supportive factors for work engagement	Job value	7
	Job Responsibility	5
	Work Interest	6
	Family Support	7
Intermediary factors between work engagement and work-life balance	Sense of Achievement	5
	Work Rewards	5
Supportive factors for work-life balance	financial security	5
	Family Support	3
	Social Status	4
Intermediary factors between work-life balance and well-being	Physical Health	6
	Mental Health	6
	Interpersonal Interaction	5

JD refers to job demands; WLB refers to work-life balance; WE refers to work engagement; WB refers to well-being.

### 4.1 Impact of job demands on work-life balance: contextual factors and mediation mechanisms

Participants articulated that factors such as job value, family support, and financial security can buffer the strain caused by job demands and promote a harmonious work-life balance. In contrast, excessive job demands may overload the teachers’ capacity, lead to physical and mental health issues, and encroach upon their personal lives, thereby thwarting work-life equilibrium.

#### 4.1.1 Contextual factors: job value, family support, and financial security

College faculty reported that job value, family support, and financial security can alleviate the negative impact of job demands on work-life balance.

**Job Value**: College faculty members who regard their work as intrinsically valuable tend to invest greater time and energy without perceiving harm to work-life harmony. Meanwhile, tasks perceived as purposeless and time-consuming are viewed as disruptive to this balance. As one participant noted,

“Excessive formalistic tasks, including burdensome paperwork and meetings, are a particular nuisance, sapping time and lacking meaning, which directly impinges on the work-life balance.” (F13).

**Family support**: Supportive families cushion the frustrations in the lives of college faculty, enabling them to better handle their work and tackle job challenges. Even with high job demands, increased family support can mitigate the negative effects on their lives, facilitating better work-life balance. Conversely, a lack of sufficient family support makes balancing work and life more difficult, especially when job pressures mount. As one participant explained,

“My parents’ assistance with childcare and my spouse’s encouragement make it feasible to maintain stability in my work-life balance, even with a demanding workload.” (M09).

**Financial security**: For college faculty, financial security encompasses adequate income, stable family circumstances, housing, and children’s education support. When these elements are assured, they counterbalance the detrimental effects of job demands on work-life balance. A secure financial footing reduces the necessity for additional work or household chores, freeing up time for priorities. Additionally, an ample salary may contribute to a perception of balanced work-life. As one participant remarked,

“Solid financial grounding spares me from daily household chores and affords me time for what truly matters” (F05).

#### 4.1.2 Mediation mechanisms: physical or mental health issues, capacity exceedance, and life disturbance

In contrast to the buffering effects of contextual factors, overwhelming job demands can exceed faculty members’ capacity, leading to physical and mental health issues, encroaching on personal life domains, and ultimately disrupting work-life balance.

**Physical Health Issues**: Intense workloads may compromise time for physical exercise, while prolonged work, particularly sedentary work, directly harms teachers’ physical health, catalyzing a state of suboptimal health that hampers work-life balance. As one participant stated,

“Mounting work stress places my physical well-being at risk. If my health is compromised, it could be a disaster for both my family and myself, affecting both work and personal life” (M11).

**Mental Health Issues**: High job demands often lead to psychological health issues because heavy workloads increase mental burden, resulting in anxiety, dissatisfaction, and complaints. If college teachers frequently experience feelings of mental exhaustion and emotional drain without addressing them, achieving work-life balance becomes challenging. Another participant observed,

“When job demands are excessively high, there is a tendency to develop a mindset of complaint and feel overwhelmed by work pressure. This psychological state will undoubtedly have a negative impact on both work and personal life.” (M10).

**Capacity Exceedance**: When job demands surpass the thresholds of college teachers’ capacity, including exceeding individual limits of ability, time, or energy, it disrupts work-life balance. As one participant described,

“In addition to occasional work overload, I also have to navigate complex interpersonal relationships due to my administrative role, as it involves interacting with various departments and coordinating multiple teams” (F07).

**Life Disturbance**: Excessive job demands erode the time and energy allocated for personal life. Furthermore, advancements in communication technology have enabled leaders to assign tasks at any time, blurring the boundaries between work and personal life. This intrusion of work into personal life disrupts work-life balance by significantly encroaching upon personal time and well-being, ultimately undermining the equilibrium between work and personal life. As one participant commented,

“High job demands, such as excessive teaching assignments and demanding research requirements, can significantly encroach upon an individual’s family life” (M09).

### 4.2 Work engagement as a catalyst for work-life balance: drivers and mediation mechanisms

Chinese college faculty reported that job value, responsibility, intrinsic work interest, and family support can enhance work engagement. Higher levels of work engagement often lead to a sense of job accomplishment. Moreover, combining these factors with appropriate reward systems can significantly contribute to achieving work-life balance.

#### 4.2.1 Drivers of work engagement: job value, job responsibility, work interest, and family support

According to college faculty, job value, job responsibility, an intrinsic interest in teaching, and family support strengthen work engagement, thereby facilitating work-life balance.

**Job Value**: College teachers perceive their teaching work as having the potential to positively transform students’ lives. They find their profession meaningful and valuable, motivating them to willingly sacrifice personal time to enhance their knowledge and capabilities for effective teaching. Consequently, even though they invest significant time and energy into their work, they do not view these commitments as detrimental to their work-life balance.

“The essence of this job lies in its potential to generate positive effects on students, and I am willing to invest time in guiding them. Despite the sacrifice of personal time, I perceive it as an acceptable trade-off considering the significance of the work.” (M03).

**Job Responsibility**: College teachers with a heightened sense of job responsibility are inclined to delve deeper into students’ learning conditions and pursue superior teaching and research outcomes. Despite the potential sacrifice of personal time, they willingly make this commitment. This dedication may contribute to enhanced teaching effectiveness and the attainment of outstanding academic accomplishments.

“In order to achieve favorable teaching outcomes, it is essential for educators to possess a thorough understanding of students’ learning situations. Personally, I ensure to diligently grade every assignment, which often requires a substantial amount of time. Consequently, I make sacrifices in terms of my personal life. Nevertheless, this dedication enables me to provide effective feedback and support for student learning.” (M08).

**Work Interest**: When the work itself brings joy to college teachers and they genuinely enjoy their profession, work becomes an inherent source of interest. As a result, even if they invest a significant amount of time in their work, it does not hinder their personal lives. This alignment between passion and occupation allows them to maintain a well-balanced lifestyle, as their work serves as a fulfilling and engaging pursuit.

“As an art teacher, I have the daily privilege of immersing myself in the realm of aesthetic beauty. Each day brings a visual feast that I find immensely pleasurable. As such, I do not experience fatigue, and I perceive a sense of balance between my work and personal life, thanks to this gratifying engagement with art.” (F05).

**Family Support**: Robust familial backing allows college faculty to fully commit to their profession, stabilizing their work-life balance. Conversely, the absence of such support may cause imbalance due to competing family obligations.

“The harmony within my family and our financial stability, along with their understanding and support towards my professional pursuits, enable me to perform well in teaching, research, and management work. As a result, I currently feel relatively balanced between work and personal life.” (M09).

#### 4.2.2 Mediation mechanisms: sense of achievement and work rewards

College faculty perceived that the sense of achievement resulting from work engagement, along with rewards received in return for their dedication, plays a significant role in fostering a sense of work-life balance.

**Sense of Achievement**: For college teachers, this sense of achievement primarily stems from their students’ progress, as their efforts contribute to students’ growth. The acknowledgment and appreciation received from students reinforce the notion that their dedication is worthwhile. Consequently, even when investing considerable time and energy into their work, they still perceive a sense of work-life balance.

“There was a moment when a student told me, ‘In your class, I felt the true essence of a university lecture’. This feedback reassured me that all the effort I put into my teaching is truly worthwhile.” (F06).

**Work Rewards**: College faculty reported that the rewards received in return for their dedication play a crucial role in fostering a sense of work-life balance. Compensation includes financial payments, tangible items, and prospects for career advancement. A discrepancy between effort exerted and compensation garnered may adversely affect college faculty’s perception of work-life equilibrium. This effect is particularly pronounced for those who take on additional responsibilities, such as serving as heads of academic departments or class supervisors. These individuals often distribute considerable energy and time into non-teaching tasks. If the rewards for these roles are inadequate, it may exacerbate their sense of imbalance between work and personal life.

“I concurrently hold the positions of department head and class supervisor, which require a significant amount of energy and effort. However, there is a substantial discrepancy between the compensation I receive and the effort I put forth, leading to a perception of imbalance between my work and personal life.” (F05).

### 4.3 From work-life balance to well-being: facilitators and mediation mechanisms

According to surveyed college faculty, financial security, family support, and perceived social status through social comparisons are pivotal in facilitating their work-life balance, which, in turn, contributes to their well-being by promoting physical and mental wellness, as well as improving interpersonal interactions.

#### 4.3.1 Facilitators of work-life balance: financial security, family support, and social status

College faculty reported that robust financial security, family support, and perceived social status through social comparisons are critical factors that facilitate work-life balance, ultimately enhancing their overall well-being.

**Financial Security**: College faculty argued that a robust financial security renders work-life balance more attainable. With financial stability, they need not sacrifice personal time for additional earning; instead, they are able to allocate adequate attention to their professional tasks, contributing to the achievement of work-life balance and subsequent elevation in well-being. Conversely, the absence of financial security complicates the pursuit of work-life balance as financial obligations might necessitate extra working hours, exerting a profound negative effect on well-being levels.

“To meet financial obligations like mortgages, auto loans, and child-rearing expenses, if the salary proves insufficient, one might be compelled to dedicate extra time to income-generating activities, which have significantly effect on well-being.” (M11).

**Family Support**: Family support encompasses the assistance and support provided by family members in terms of sharing time commitments, taking on responsibilities, and handling challenges. Those educators receiving substantial family support tend to report an enhanced experience of work-life balance and thus greater well-being. Conversely, a lack of such support may aggravate work-life imbalances and lower their well-being.

“When facing an imbalance between work and personal life, such as when work encroaches upon my personal life, I can still experience a sense of well-being if I receive ample family support during such times.” (M03).

**Social Status**: Perceived social status through social comparison involves college teachers evaluating their circumstances against their peers in other institutions or sectors, which can generate feelings of either satisfaction or dissatisfaction. Those who perceive their social status as elevated and experience their job roles as less taxing or more autonomous compared to others are inclined to feel a sense of work-life balance. Despite the high pressure experienced by college teachers in first-tier and second-tier cities, as well as those in prestigious universities categorized under the 985 and 211 projects, their elevated social status contributes to their perception of work-life balance and heightened well-being.

“The general social prestige of university educators is relatively high; this status brings psychological equanimity and a greater sense of well-being.” (M10).

#### 4.3.2 Mediation mechanisms: physical or mental health, and interpersonal harmony

According to the participants, work-life balance significantly enhances their well-being by preserving physical health, fostering mental wellness, and promoting harmonious interpersonal interactions.

**Physical Health**: Participants argued that maintaining work-life balance positively impacts physical health, which correlates with increased well-being. In contrast, an imbalance between work and life may lead to disorder across various aspects of life, potentially resulting in neglected physical activity and deteriorating physical health, thereby decreasing overall well-being.

“Work-life balance among college faculty exhibits higher work efficiency and greater life satisfaction, allowing them to allocate sufficient time for physical exercise. This in turn contributes to improved physical health and a heightened sense of well-being.” (M01).

**Mental Health**: Participants commonly reported that work-life balance alleviates stress from both professional and personal spheres while fostering positive emotions, enhancing psychological well-being, and ultimately improving overall well-being. Among college teachers with good work-life balance, they experience less work-related stress, fewer negative emotions at work, enjoy harmonious family relationships, and face fewer worries or concerns. This balance contributes to higher work efficiency, providing them with time to enjoy their lives and maintain stable psychological well-being, thereby enhancing overall well-being.

“From my perspective, educators with a balanced work-life exhibit high efficiency, which allows them ample leisure time to enjoy their personal lives. As a result, they are more likely to experience better mental well-being and ultimately higher overall well-being.” (F12).

**Interpersonal Harmony**: Good interpersonal interactions, spanning relations with superiors, colleagues, students, and family, are identified by college teachers as one of the benefits derived from work-life balance. When they achieve work-life balance, they typically exhibit positive emotions and maintain a calm mindset, enabling them to engage in pleasant interactions and foster harmonious interpersonal relationships. Consequently, they tend to experience an elevated sense of well-being. Conversely, an imbalance between work and personal life can easily give rise to negative mental states and emotions, which may result in poor interpersonal relationships and lower levels of well-being.

“An imbalance can prompt the transference of negativity to work or home environments, potentially souring interactions with superiors, peers, students, and family, which consequently erodes one’s well-being” (F07).

## 5 Discussion

### 5.1 Theoretical advancements to the work-life balance model

**Figure 1:**
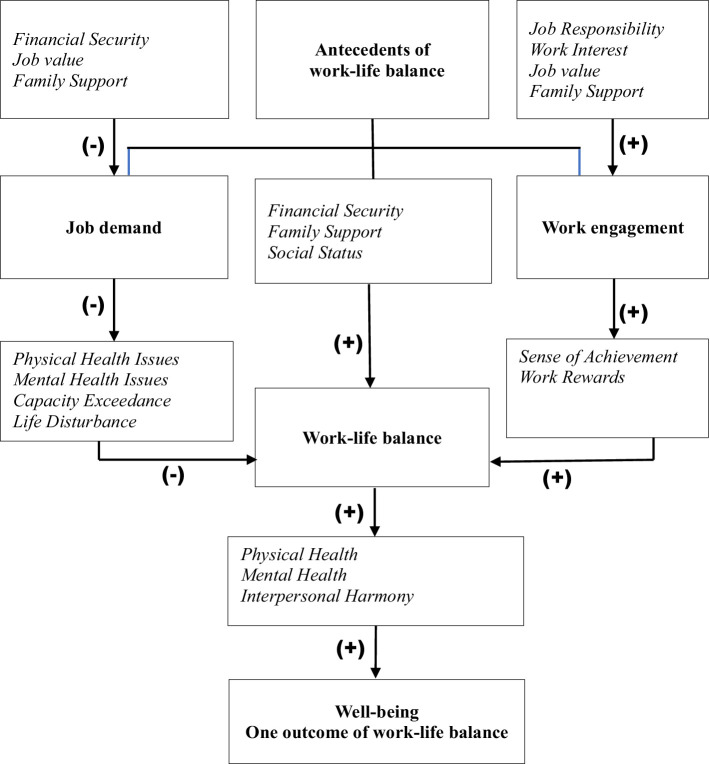
The mechanism of work-life balance affecting well-being. The **bold** text highlights the antecedent variables (job demand, work engagement) and the outcome variable (Well-being) in the original work-life balance model. The **italicized** text represents the new influencing factors added to the original model based on the research findings. These factors include those that influence job demand and work engagement, those generated by job demand and work engagement that affect work-life balance, and those resulting from work-life balance itself that in turn influence Well-being. The symbol “-” denotes negative effects, while “+” indicates positive effects.

The traditional work-life balance model emphasizes organizational factors (e.g., job demands) and individual factors (e.g., work engagement) as antecedents of work-life balance (12). However, this study significantly expands upon the existing model by incorporating newly defined relationships and mechanisms, which are highlighted in Figure 1. This expanded diagram provides a more explicit illustration of how antecedent variables influence job demands, work engagement, and ultimately work-life balance and well-being through various mediating mechanisms. By providing new insights into the interplay among variables within the work-life balance model, this study not only deepens the theoretical understanding but also paves the way for practical applications in policy and institutional reforms.

#### 5.1.1 Nonlinear dynamics of job demands and work-life balance

Previous research has consistently acknowledged that job demands can hinder work-life balance by interfering with family life. For instance, such demands have been shown to diminish college teachers’ family engagement (57) and amplify work–family conflicts (58). Building on this foundation, the current study, situated within the context of Chinese higher education institutions, revealed that the relationship between job demands and work-life balance is not simply linear. Specifically, the study identified financial security, job value, and family support as antecedent variables that can mitigate the adverse impact of job demands on work-life balance. Furthermore, mediating variables such as physical and mental health issues, capacity exceedance, and life disturbance were found to intensify the negative effects of job demands on work-life balance. These findings not only clarify the facilitating or inhibiting roles of these factors on work-life balance but also elucidate the complex interplay between job demands and work-life balance, thereby offering novel insights into this dynamic relationship.

#### 5.1.2 Nonlinear dynamics of work engagement and work-life balance

Management research has confirmed that supportive work arrangements, including executive and peer backing, family-oriented methods (59), human resource protocols (60), and empowering leadership (61), can enhance the positive impact of work engagement on work-life balance. Extending this line of inquiry, the current study, conducted in the specific context of Chinese higher education institutions, revealed that the relationship between work engagement and work-life balance is not linear. Specifically, the study identified job responsibility, work interest, job value, and family support as key antecedent variables that strengthen the positive impact of work engagement on work-life balance. Additionally, mediating variables such as a sense of achievement and work rewards were found to foster a sense of work-life balance. These findings underscore the importance of considering both antecedent and mediating variables in understanding the complex dynamics between work engagement and work-life balance.

#### 5.1.3 Mechanisms underpinning work-life balance and well-being

Existing literature has demonstrated that familial discord (14) and intense job demands (62) can disrupt work-life balance, subsequently diminishing well-being. Conversely, the presence of managerial support and familial solidarity (63), along with an organizational culture sensitive to family needs (64), can facilitate work-life balance, thereby enhancing well-being. Furthermore, evidence suggests that a harmonious work-life balance can amplify the positive effects of educator-student rapport (65) and overall quality of life (66) on well-being while mitigating the detrimental impacts of gender disparities, occupational stress, and unhealthy workplace environments (15). Anchored in the context of Chinese higher education institutions, the current study revealed that the relationship between work-life balance and well-being is not straightforwardly linear. Drawing on the experiences of Chinese college faculty, the study identified physical and mental health, and interpersonal harmony as mediating variables that promote work-life balance. The benefits of such equilibrium, in turn, enhance overall well-being. These findings highlight the multifaceted nature of the relationship between work-life balance and well-being, emphasizing the need for a comprehensive approach to understanding and improving well-being in the workplace.

### 5.2 Practical recommendations for policy and institutional reforms

#### 5.2.1 Empowering faculty through personal and professional growth

To foster sustainable personal and professional development, university teachers should enhance their sense of identification with and commitment to the higher education profession. This can be achieved by improving their perceived job value and responsibility, as well as by cultivating a passion for teaching and research. Actively engaging in professional development can drive career growth. Additionally, maintaining positive relationships with family members, colleagues, supervisors, and students is crucial for securing necessary support in both personal and professional spheres. Furthermore, prioritizing physical exercise and mental health ensures holistic well-being. Collectively, these practices can contribute to sustainable personal and professional development, ultimately enhancing work-life balance and well-being.

#### 5.2.2 Strengthening organizational and administrative competencies

Academic institutions are encouraged to strengthen their organizational and administrative competencies. This includes enhancing working conditions, task structures, management frameworks, and evaluation methodologies. By doing so, institutions can amplify the perceived value of tasks, enabling college faculty to better appreciate the significance of their roles in teaching, research, service, and administration. Institutions should also establish judicious organizational mechanisms that effectively utilize modern office technologies and delineate clear boundaries between work and personal life to prevent excessive work intrusions. These measures can significantly enhance work-life balance and overall job satisfaction among faculty members.

#### 5.2.3 Supporting faculty well-being through health promotion and work-life balance

Providing college faculty members with adequate compensation is essential to alleviate survival pressures. It is equally important to reduce job demand-induced disruptions to work-life balance and to enhance the positive effects of work engagement on this balance. Focusing on the overall well-being of faculty members is crucial and can be achieved through regular health check-ups and psychological evaluations. Moreover, fostering constructive interactions among faculty, students, and the broader academic community is vital. Collectively, these measures pave the way for achieving professional fulfillment and a harmonious work-life balance, ultimately leading to elevated levels of well-being. By implementing these recommendations, institutions can create a supportive environment that promotes both professional success and personal well-being among faculty members.

## 6 Conclusion and research limitations

### 6.1 Conclusion

As suggested by college faculty members, perceived job value, family support, and financial security can alleviate the pressures of job demands, which contribute to a harmonious work-life balance. On the other hand, overwhelming job demands may overload the teachers’ capability, precipitate health problems, or encroach upon personal life, thus hindering the attainment of work-life balance.

Moreover, teachers’ perceived job value, job responsibility, an intrinsic interest in teaching, and family support are factors that promote work engagement. This, in turn, enhances a sense of job accomplishment, which, if augmented with corresponding work rewards, facilitates the achievement of work-life balance.

Furthermore, robust financial security, adequate family support, and perceived social status through social comparisons are identified by college faculty members as critical factors in facilitating work-life balance, which in turn, contributes to overall well-being by promoting physical and mental wellness, as well as by improving interpersonal interactions.

### 6.2 Limitations and future research recommendations

Firstly, the study’s sample lacks university teachers from China’s northeastern region, which compromises its national representativeness. Given that China is divided into four major economic regions (Eastern, Central, Western, and Northeastern), each with unique economic and educational characteristics, this omission is significant. The absence of participants from the Northeastern region limits our ability to generalize the findings across all regions of China. Secondly, the lack of cross-cultural research involving teachers from other countries further restricts the generalizability of our findings. To enhance the validity and broader applicability of our research, future studies should employ stratified sampling methods to ensure balanced representation across all economic zones. Furthermore, by establishing collaborations with local universities in those underrepresented regions and increasing cross-cultural research, we aim to make our findings more universally applicable. Specifically, such collaborations will facilitate access to diverse participant pools, while cross-cultural research will allow for a richer comparison across different contexts.

Secondly, due to the COVID-19 pandemic, this study utilized online interviews to investigate college teachers. While online interviews offer several advantages, such as reducing the risk of infection and facilitating data processing through audio and video recordings (67), they also present notable limitations. Specifically, online interviews may restrict the researcher’s ability to observe non-verbal cues, which are often critical for understanding participants’ emotional states and responses. This limitation is particularly pronounced when interviewees are unwilling or unable to participate via video, potentially leading to missing information. Moreover, technical issues such as unstable internet connections or platform malfunctions can disrupt the interview process, affecting both the quality of data collected and the participant’s comfort level. These factors may influence the depth and richness of the responses obtained. To mitigate these limitations, future research should consider a hybrid approach that combines face-to-face and online interviews. Face-to-face interviews allow researchers to observe non-verbal behavior and record immediate reactions, enabling more in-depth follow-up questions based on the research questions (68). This combination can enhance the validity and reliability of the findings by providing a richer dataset and compensating for the potential shortcomings of online-only methods.

Thirdly, this study did not explore the differences in the impact of job demand, work engagement, work-life balance, and well-being among college teachers of different genders, ages, and professional titles. To maintain conciseness, the presentation focused on exploring the views of Chinese college teachers regarding the impact of job demand, work engagement, and work-life balance on well-being. Therefore, future research could further analyze the influence of gender, age, and professional title on the relationships among job demand, work engagement, work-life balance, and well-being among Chinese college teachers. Additionally, it would be valuable to investigate their perspectives on the reasons and mechanisms through which job demand and work engagement impact well-being, as well as the factors and processes through which work-life balance affects well-being.

## Data Availability

The raw data supporting the conclusions of this article will be made available by the authors, without undue reservation.
